# Supplementary material for: The Development and Psychometric Properties of Malay Language Child Oral Health Impact Profile—Short Form 19 (ML COHIP-SF 19)
Source: Healthcare (Basel). 2025 Jan 28;13(3):257. doi: 10.3390/healthcare13030257 (PMC11817817; doi:10.3390/healthcare13030257)
Supplement: Supplementary file 1 [file healthcare-13-00257-s001.zip › healthcare-3386102-supplementary.pdf]

Supplementary S1: COHIP-SF 19 English and Malay language items

	**English**	**Malay language**
	**In the past three months, how often have you?**	Dalam masa 3 bulan lepas, berapa kerapkah anda?
**1.**	**Had pain in your teeth/ toothache**	Mengalami sakit gigi
**2.**	**Had crooked teeth or spaces between your teeth**	Mempunyai gigi yang tidak tersusun atau gigi jarang
**3.**	**Had discoloured teeth or spots on your teeth**	Mempunyai gigi yang berbeza warna atau bertompok 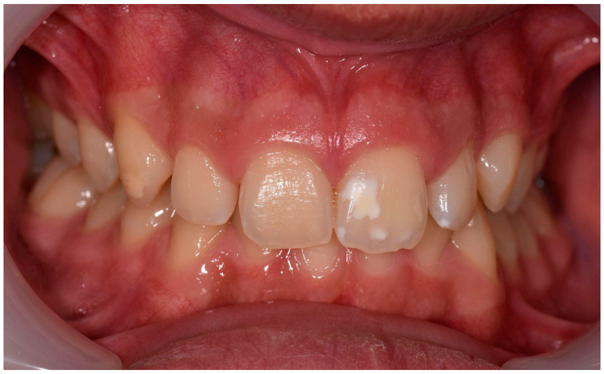
**4.**	**Had bad breath**	Mengalami masalah nafas berbau
**5.**	**Had bleeding gums**	Mengalami masalah gusi berdarah
**6.**	**Been unhappy or sad because of your teeth, mouth, or face**	Berasa tidak gembira atau sedih disebabkan oleh gigi, mulut atau muka anda
**7.**	**Missed school for any reason because of your teeth, mouth, or face**	Tidak hadir ke sekolah atas sebarang alasan yang berkaitan dengan gigi, mulut atau muka anda
**8.**	**Been confident because of your teeth, mouth, or face**	Berasa yakin (‘confident’) disebabkan oleh gigi, mulut atau muka anda
**9.**	**Had difficulty eating foods you would like to because of your teeth, mouth, or face**	Susah untuk makan disebabkan oleh gigi, mulut atau muka anda
**10.**	**Felt worried or anxious because of your teeth, mouth, or face**	Berasa bimbang atau risau disebabkan oleh gigi, mulut atau muka anda

11.	**Not wanted to speak/ read out loud in class**	Tidak mahu bercakap atau membaca di khalayak ramai di dalam kelas
12.	**Avoided smiling or laughing with other children because of your teeth, mouth, or face**	Mengelak dari senyum atau ketawa bersama kanak-kanak lain disebabkan oleh gigi, mulut atau muka anda
13.	**Had trouble sleeping because of your teeth, mouth, or face**	Susah untuk tidur disebabkan oleh gigi, mulut atau muka anda
14.	**Been teased, bullied or called names by other children because of your teeth, mouth, or face**	Diejek, dibuli atau digelar menggunakan panggilan nama yang tidak elok oleh kanak-kanak lain disebabkan oleh gigi, mulut atau muka anda
15.	**Felt that you were attractive (good looking) because of your teeth, mouth, or face**	Berasa diri anda menarik (cantik/ kacak) disebabkan oleh gigi, mulut atau muka anda
16.	**Felt that you look different because of your mouth, teeth, or face**	Berasa diri kelihatan berbeza disebabkan oleh gigi, mulut atau muka anda
17.	**Had difficulty saying certain words**	Susah untuk menyebut perkataan-perkataan tertentu
18.	**Had difficulty keeping your teeth clean**	Susah untuk memastikan gigi anda sentiasa bersih
19.	**Been worried about what other people think about your teeth, mouth, or face**	Berasa risau tentang pendapat orang lain terhadap gigi, mulut atau muka anda
	***Response categories: “never”, “almost never”, “sometimes”, “fairly often”, and “almost all the time”**	*Response categories: “tidak pernah”, “hampir tidak pernah”, “kadang-kadang”, “agak kerap”, and “hampir setiap masa”
	**Overall, please rate your oral health**	Sila nilai kesihatan mulut anda secara keseluruhannya
	***Response categories: “poor”, “fair”, “average”, “good”, and “excellent”**	*Response categories: “teruk”, “memuaskan”, “sederhana”, “baik”, and “cemerlang”
